# Topological transformations in proteins: effects of heating and proximity of an interface

**DOI:** 10.1038/srep39851

**Published:** 2017-01-04

**Authors:** Yani Zhao, Mateusz Chwastyk, Marek Cieplak

**Affiliations:** 1 Institute of Physics, Polish Academy of Sciences, Al. Lotników 32/46, 02-668 Warsaw, Poland

## Abstract

Using a structure-based coarse-grained model of proteins, we study the mechanism of unfolding of knotted proteins through heating. We find that the dominant mechanisms of unfolding depend on the temperature applied and are generally distinct from those identified for folding at its optimal temperature. In particular, for shallowly knotted proteins, folding usually involves formation of two loops whereas unfolding through high-temperature heating is dominated by untying of single loops. Untying the knots is found to generally precede unfolding unless the protein is deeply knotted and the heating temperature exceeds a threshold value. We then use a phenomenological model of the air-water interface to show that such an interface can untie shallow knots, but it can also make knots in proteins that are natively unknotted.

A closed curve may form a well-defined mathematical knot whose main characteristic is the number of intersections when projected onto a plane. Unknotting it would require slicing through it. A circular DNA forms a closed curve that typically contains knots. There have been many studies of knots in such DNA^1^,^2^,^3^,^4^. DNA, however, may exist both in closed and open forms, but in either case all topological transformations must occur through the action of cutting and reattaching enzymes such as topoisomerases^5^,^6^,^7^ and resolvases^8^,^9^. The cutting has been observed to be facilitated by supercoiling that tightens DNA knots^10^. Topology changes of open DNA also require cutting because of the large size of the molecule.

Knotted proteins^11^,^12^,^13^,^14^,^15^,^16^,^17^,^18^, on the other hand, are small compared to DNA and their topological states may evolve in time through large conformational changes such as folding from an unknotted extended state and unfolding. All of the native protein knots can be obtained by repeatedly twisting a closed loop and then threading one of the ends through the loop. Therefore, they are called twist knots.

Theoretical studies have established that the folding behavior depends on whether the native state of the protein is knotted in a deep or shallow fashion: it is much harder to tie the former than the latter^19^,^20^,^21^,^22^,^23^,^24^,^25^ but the process, in both cases, is predicted to be helped by the nascent conditions provided by the ribosomes^24^,^25^. A protein is considered to be deeply knotted in its native state if both ends of the knot, as determined, *e.g.* by the KMT algorithm^11^,^26^, are far away from the termini (in practice, by more than about 10 residues). Otherwise it is considered to be knotted shallowly. Notice that the sequential heterogeneity of a protein positions the knot in a specific sequential region and tightening of the knot, upon protein stretching from its termini, goes through jumps to specific locations^27^.

In this paper, we consider two different types of conformational changes: thermal unfolding and protein deformation induced by a nearby air-water interface. We find that a sufficiently high temperature can untie any type of knots if one waits long enough, but the topological pathways of unfolding are generally not the reverse of those found for folding. The air-water interface may induce unknotting of shallow knots but we have also found an example of a situation in which a protein acquires a knot. We perform our simulations within a structured-based coarse-grained model and the interface is introduced empirically through coupling of a directional field to the hydropathy index of an amino acid residue in the protein^28^. Such a field favors the hydrophilic residues to stay in bulk water and the hydrophobic residues to seek the air, leading to surface-induced deformation and sometimes even to denaturation, defined by the loss of the biological functionality. The simplified character of the model leads to results that are necessarily qualitative in nature – they just illustrate what kinds of effects the presence of the interface may bring in, especially in the context of the topological transformations.

It should be noted that the behavior of proteins and protein layers at the air-water interface is of interest in physiology and food science. For instance, the high affinity of lung surfactant proteins to stay at the surface of pulmonary fluid generates defence mechanisms against inhaled pathogens^29^. The layers of the interface-adsorbed proteins typically show viscoelastic properties^30^,^31^,^32^ and the enhanced surface viscosity of the pulmonary fluid is thought to provide stabilization of alveoli against collapse^33^. Protein films in saliva increase its retention and facilitate its functioning on surfaces of oral mucosa^34^. Various proteins derived from malted barley have been found to play a role in the formation and stability of foam in beer^35^. Adsorption at liquid interfaces has been demonstrated to lead to bending of and ring formation in amyloid fibers^36^.

There are many theoretical questions that pertain to the behavior of proteins at the air-water interfaces. The one that we explore here is whether the interfaces can affect topology. We find that indeed it can: the shallowly knotted proteins may untie and some unknotted proteins may acquire a shallow knot. Deeply knotted proteins get distorted but their knottedness remains unchanged. We consider four proteins: (1) the deeply knotted YibK from *Haemophilus influenzae* with the PDB^37^ structure code 1J85^38^, (2) the shallowly knotted MJ0366 from methanogenic archea *Methanocaldococcus jannaschi* (PDB:2EFV) – this is the smallest knotted protein known, (3) the shallowly knotted DndE from *Escherichia coli* (PDB:4LRV), and (4) chain A of the pentameric ligand-gated ion channel from *Gleobacter violaceus* (PDB:3EAM)^39^ which is an unknotted protein. From now on, we shall refer to these proteins by their PDB codes. In order to elucidate the effects of hydrophobicity we shall also consider certain “mutated” sequences in which certain residues are replaced by other residues without affecting the native structure. The proteins 1J85, 2EFV, and 4LRV have the sequential lengths of 156, 82 and 107 respectively and the corresponding sequential locations of their knots are 78–119, 11–73, and 8–99. Thus the knot in 2EFV is shallow at the C-terminus whereas the one in 4LRV – at both termini.

## Methods

Our basic structure-based coarse-grained model of a protein has been described in detail in refs 40, 41, 42 and 43. We use our own code. Other implementations of structure-based (or Go-like^44^) models can be found in refs 45, 46, 47, 48, 49 and 50. The primary ingredient of the model is the contact map which specifies which residues may form non-bonding interactions described by a potential well. There are many types of contact maps, as summarized in ref. 51, and we take the one denoted by OV here. This OV map is derived by considering overlaps between effective spheres assigned to heavy atoms in the native state. The radii are equal to the van der Waals radii multiplied by 1.24^52^. The potentials assigned to the contacts between residues *i* and *j* are given by


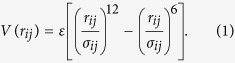


The length parameters *σ*_*ij*_ are derived pair-by-pair from the native distances between the residues – the minimum of the potential must coincide with the *α*-C − *α*-C distance. Consecutive *α*-C atoms are tethered by the harmonic potential 
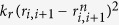
, where *k*_*r*_ = 100 *ε*/Å^2^ and 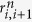 is the native distance between *i* and *i* + 1. The local backbone stiffness favors the native sense of the local chirality, but using the self-organized polymer model^53^ without any backbone stiffness yields similar results^43^.

The value of the parameter *ε* has been calibrated to be of order 110 pN Å which was obtained by making comparisons to the experimental data on stretching^42^. We use the overdamped Langevin thermostat and the characteristic time scale in the simulations, *τ*, is of order 1 ns. The equations of motion were solved by the 5th order predictor-corrector method. Due to overdamping, our code is equivalent to the Brownian dynamics approach. A contact is considered to be established if its length is within 1.5 *σ*^41^. The trajectories typically last for up to 1 000 000 *τ*.

Despite its simplicity, the structure-based model used here has been shown to work well in various physical situations. In particular, it is consistent (within 25% error bars) with the experimental results on stretching for 38 proteins^41^,^42^,^46^. It also has good predictive powers. For instance, our simulations^41^ have yielded large mechanostability of two cellulosome-related cohesin proteins c7A (PDB:1AOH) and c1C (PDB:1G1K) that got confirmed experimentally^54^. In the case of c7A, the calculated value of the characteristic unravelling force is 470 pN and measured – 480 pN. The model also reproduces the intricate multi-peak force profile corresponding to pullling bacteriorhodopsin out of a membrane^55^. The equilibrium positional RMSF patterns have been found to be agree with all-atom simulations, for instance, for topoisomerase I^56^ and Man5B complexed with a hexaose^57^. This model has also been used to study nanoindentation of 35 virus capsids^58^,^59^ and to demonstrate that characteristic collapse forces and the initial elastic constants are consistent with the experimental data^60^.

The air-water interface is centered at *z* = 0 and extends in the *x* − *y* plane so that the bulk water corresponds to negative *z* and air to positive *z*. However, it should be noted that the interface is diffuse – its width is denoted by *W*. The interface-related force acting on the *i*th *α*-C atom is given by^28^


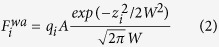


where *q*_*i*_ is the hydropathy index, *A* is set equal to 10 *ε*, and *W* = 5 Å. We use the values of *q*_*i*_ as determined by Kyte and Doolittle^61^. They range between −4.5 for the polar and charged ARG and 4.5 for the hydrophobic ILE. Other possible scales are listed in ref. 62. For each protein, we can identify a degree of hydrophobicity *H* in terms the values of *q*_*i*_ of its amino acids, 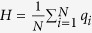, where the sum is over the amino acids in the protein. Properties of protein conformations are assessed by the fraction, *Q*, of the native contacts that are present in the conformation.

The phenomenologically motivated addition of the air-water term to the basic structure-based model leads to the experimentally observed formation of a protein layer^63^ at the interface and gives rise to the in-layer diffusive behavior which is characteristic of soft colloidal glass with the intermediate values of the fragility indices^28^. Specifically, as a function of the number density of the proteins at the interface, the surface diffusion coefficient obeys a Vogel-Fulcher-Tamann law. This is consistent with the microrheology experiments on the viscoelastic behavior of protein layers^64^,^65^.

In the initial state, *N*_*p*_ proteins (*N*_*p*_ is between 2 and 50 are placed in a large square box so that their center of mass are around *z* = −3.2 ± 0.4 nm with the *x* and *y* coordinates selected randomly. Their initial conformations are native. The box is bounded by a repulsive bottom at −7 nm and by repulsive sides. The force of the wall-related repulsion decays as the normal distance from the wall to the tenth power. The walls may be brought to a desired smaller separation in an adiabatic way, however, here we focus on the dilute limit in which the proteins are far apart. The purpose of considering many proteins simultaneously is to generate statistics of behavior and a spread in the arrival times to the interface. If the proteins happen to come close to one another, their mutual interactions correspond to repulsion that forbids overlap.

The thermodynamic properties of the system in the bulk are assessed by determining the temperature (*T*) dependence of *P*_0_ – the probability of all contacts being established simultaneously. *P*_0_ is determined in several long equilibrium runs. For typical unknotted proteins, the optimum in the folding time is in the vicinity of *T* = *T*_*r*_ = 0.3 *ε*/*k*_*B*_ (*k*_*B*_ is the Boltzmann constant) where *P*_0_ is nonzero. A more detailed discussion of this point is presented in ref. 43. *T*_*r*_ then plays the role of the effective room temperature. This value of *T* is also consistent with the calibration of the parameter *ε*.

Thermal unfolding is studied by considering a number of trajectories at *T* > *T*_*r*_ that start in the native state and last for up to 1 000 000 *τ*. Unfolding is achieved if all native contacts that are sequentially separated by more than a threshold value of *l* residues are ruptured for the first time^24^. An ideal unfolding would involve breaking of all contacts, but such simulations would take unrealistically long to run. We thus introduce the threshold that separates contacts that are sequentially local from the non-local ones. Contact in *α*-helices do not exceed the distance of 4. Usually, we take *l* = 10. The median value of this rupture time defines the characteristic and *l*-dependent unfolding time *t*_*unf*_. An alternative criterion could involve crossing a threshold value of *Q*.

The dynamics of staying in the knotted state in the bulk or on approaching the air-water interface is assessed by monitoring the time dependence of *P*_*k*_(*t*) – the probability that, at time *t*, the protein stays in its native topology.

## Results

### Thermal unfolding

Even though the deeply knotted 1J85 protein is difficult to fold from a fully extended conformation at any *T*, we find that it is easy to unfold it at elevated *T*, if the waiting time is sufficiently long. Within our cutoff-time, we could observe it happen for *T* ≥ 0.85 *ε*/*k*_*B*_. For *T* ≥ 1.0 *ε*/*k*_*B*_ we have not recorded any refolding events after full unfolding. At *T* = 0.85 *ε*/*k*_*B*_, 21% of the 28 trajectories resulted in retying the trefoil knot. Note that the starting conformation for the refolding process is not at all fully extended and is thus biased towards knotting – a situation most likely encountered in ref. 20. The loss of all contacts may result in conformations that look like expanded globules. Taking *l* of 10, the values of *t*_*unf*_ are 565 045, 116 512, and 25 479 *τ* for *T* equal to 0.9, 1.0, and 1.2 *ε*/*k*_*B*_ respectively. Breaking contacts is not directly related to untying. We find that the median untying times are 198 850, 85 050, and 34 710 *τ* for the same temperatures respectively. This indicates that at the lower two of the three temperatures untying precedes unfolding and decreasing the *l* enhances the gap between the two events (see Fig. S1 in Supplementary Information, SI). Only in one trajectory out of the total of 25 at *T* = 1.0 *ε*/*k*_*B*_, unfolding takes place 200 *τ* earlier than untying. For *T* = 1.2 *ε*/*k*_*B*_, unfolding takes place before untying in most of the trajectories if one takes *l* = 10, but for *l* = 4, the reverse holds. It is only at *T* = 1.5 *ε*/*k*_*B*_ that unfolding always takes place before untying even if *l* = 4.

The unfolding pathway of knotted proteins has been studied in ref. 66 for a structurally homologous YibK-like methyltransferase (PDB:106D). The theoretical part of the study also involved a structure-based model, but with a very different contact map. The finding was that untying takes place after unfolding and this was taken as a signature of a certain hysteresis in the process. However, the value of *T* was not specified – presumably the simulations were done at a high *T*. We just demonstrate that the actual sequence of the unfolding events depends on *T*. Since, in our model, *T* of 1.0 *ε*/*k*_*B*_ corresponds to about 850 K, it is the still lower *T* that are relevant experimentally and thus observing unfolding before unknotting on heating seems unlikely. However, the experimental studies involve chemical denaturation by Gnd-HCl, which allows for a broader range of conditions that are meaningful experimentally.

**Figure 1:**
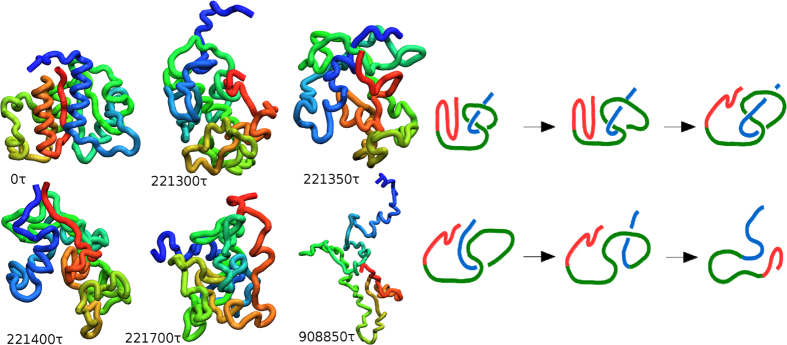
An example of thermally induced unfolding of 1J85 through the DT mechanism at 0.85 *ε*/*k*_*B*_. The six panels on the left show successive snapshots of the backbone conformations at times indicated. The six panels on the right provide the corresponding schematic representations of these conformations. The N-terminal segment is shown in shades of orange and red, the C-terminal segment in shades of blue, and the middle segment in shades of green.

**Figure 2:**
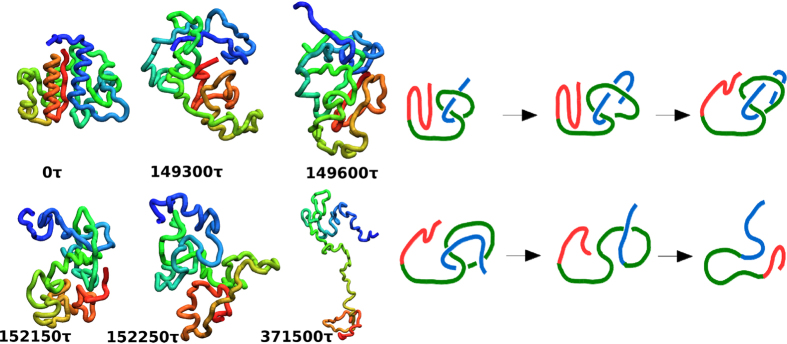
Similar toFig. 1, but unfolding proceeds through the SK mechanism and the trajectory corresponds to *T* = 0.9 *ε*/*k*.

The mechanisms of unknotting in 1J85 are dominated by direct threading (DT) events, illustrated in Fig. 1, followed in statistics by slipknotting (SK) events, as illustrated in Fig. 2. We observe no other unfolding mechanisms. They have been discussed in refs 20, 25 in the context of folding except that now they operate in reverse. For instance, the DT mechanism involves pulling of a terminus of the protein out of a loop and the SK mechanism involves pulling a slipknot out of the loop. The determination of the precise nature of the process is based on a visual monitoring of the subsequent snapshots of the evolution. The exact proportions between the mechanisms depend on the *T*. The red color is used for the N-terminal segment, blue – for the C-terminal one and green – for the middle part of the backbone. However, the number of instances of unknotting through SK decreases with a growing *T* (32%, 8%, and 0% at 0.9, 1.0 and 1.2 *ε*/*k*_*B*_). Unknotting in the trajectory shown in Fig. 1 takes place at time 221 400 *τ* so the last panel corresponds to a situation in which the protein is unknotted but not yet fully unfolded.

Topological pathways of folding in the shallowly knotted 2EFV have been demonstrated to be of two basic kinds: through single loops^22^ or through two smaller loops^25^. The latter is the dominant pattern and is a two-stage process. The two-stage pathways have not been observed in the deeply knotted 1J85. In each of these cases, the specific mechanisms of making the knot involve, in various proportions, DT, SK, and mouse-trapping (MT). MT is similar to DT but the knot-loop moves onto the terminal segment of the protein instead of the other way around. There is also a possibility of an embracement (EM)^25^ in which a loop forms around a terminal segment. The DT, SK, MT, and EM mechanisms may operate either at the level of a single larger loop in a process, which is topologically one-stage, or at the level of two smaller loops and hence in two stages. Again, the identification of the nature of the pathway is obtained visually.

**Figure 3:**
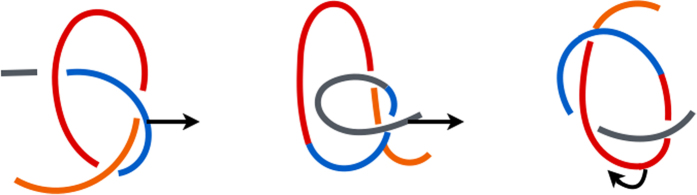
A schematic representation of a single-loop DT (left), SK (middle) and MT-based (right) thermal unfolding of 2EFV at *T* = 0.7 *ε*/*k*_*B*_.

When unfolding 2EFV at *T* = 0.5 *ε*/*k*_*B*_, all events are two-stage, exclusively SK-based, and are soon followed by refolding. At 0.7 *ε*/*k*_*B*_, only 28% of 50 trajectories are two-stage (DT and SK are involved in each stage) and the remaining ones are one-stage. Most of them refold back soon afterwards. At *T* ≥ 1.0 *ε*/*k*_*B*_ there is no refolding and all trajectories unfold through the single loop mechanism. The process is dominated by the DT events, followed by SK, and then some MT ones. An example of a DT-based pathway is shown in Fig. 3. The N-terminal segment (1–16) is marked in orange, sites 17–53 in red, sites 54–78 in blue, and the C-terminal segment (79–82) in gray. In all trajectories, untying of 2EFV occurs before thermal unfolding (for 4 ≤ *l* ≤ 10 at *T* ≤ 1.5 *ε*/*k*_*B*_ – see Fig. S1 in SI).

The physics of folding and unfolding in 4LRV is found to be similar to that of 2EFV, but the DT unfolding events are more likely to proceed from the N-terminus instead of the C-terminus. Another difference is that folding at *T*_*r*_ is seen to take place exclusively through the two-loop mechanism. 12 out of 50 trajectories led to folding. 7 of them proceeded through the EM-SK pathway, 4 through SK-SK, and 1 through DT-SK (see Fig. S2 in SI).

We conclude that the thermal unfolding processes of the knotted proteins are generally distinct from a simple reversal of folding. For instance, the dominant two-loop folding trajectories do not form a reverse topological template for the dominant single-loop unfolding trajectories. A similar observation has been already made for unknotted proteins although it involves no changes in the topology^67^.

### Knot-untying by the air-water interface

**Figure 4:**
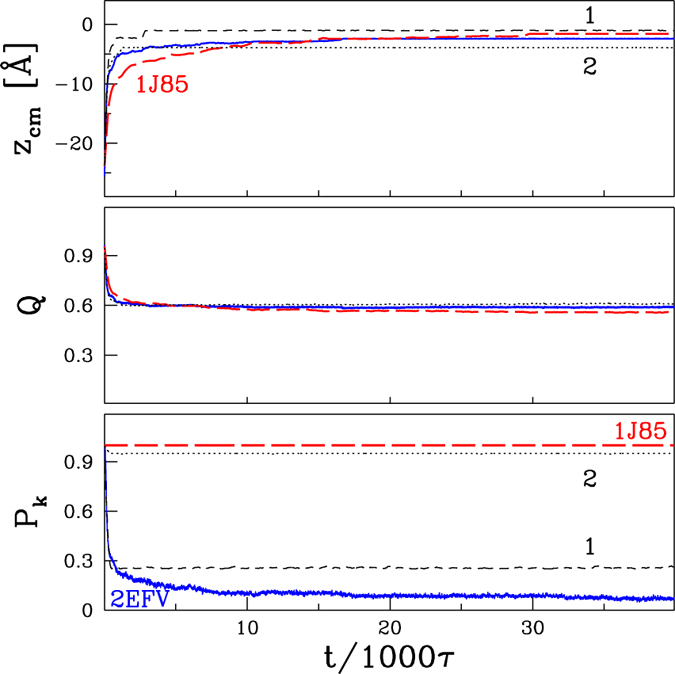
Distance to the surface (*z*_*cm*_), fraction of preserved native contacts (*Q*) and probability of being knotted (*P*_*k*_) as a function of time, *t*, for 1J85 (red), 2EFV (blue) and its two mutants (1,2; black) at the air-water interface. The data are based on one trajectory in each case.

**Figure 5:**
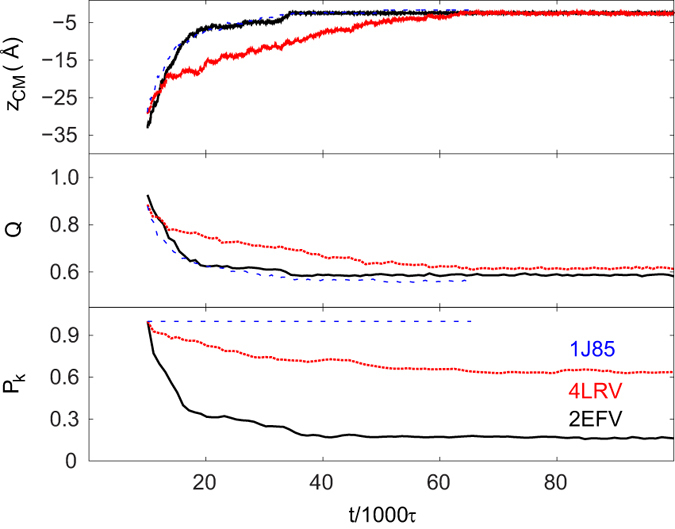
Time-evolution of *z*_*cm*_, *Q* and *P*_*k*_ averaged over 50 proteins at *T* = *T*_*r*_. The black, red, and blue lines are for 2EFV, 4LRV, and 1J85 respectively. During the first 10 000 *τ*, the proteins diffuse around without the interface.

We now consider the interface-related effects at *T* = *T*_*r*_. The proteins that come to the interface get deformed and lose some of their native contacts. We find that these phenomena do not affect the topological state of the deeply knotted 1J85 as demonstrated in Fig. 4. The data shown are for one example trajectory which corresponds to a specific starting protein orientation with respect to the interface. Various orientations and different initial locations yield various adsorption times. When one averages over 50 proteins, one gets the results shown in Fig. 5. The loss of contacts is related to the approach of the center of mass of the protein(s) to the center of the interface. The knot-ends may shift from one trajectory to another, but there is no knot untying. Furthermore combining the effects of the interface with those of an elevated temperature is found not to promote any untying.

**Figure 6:**
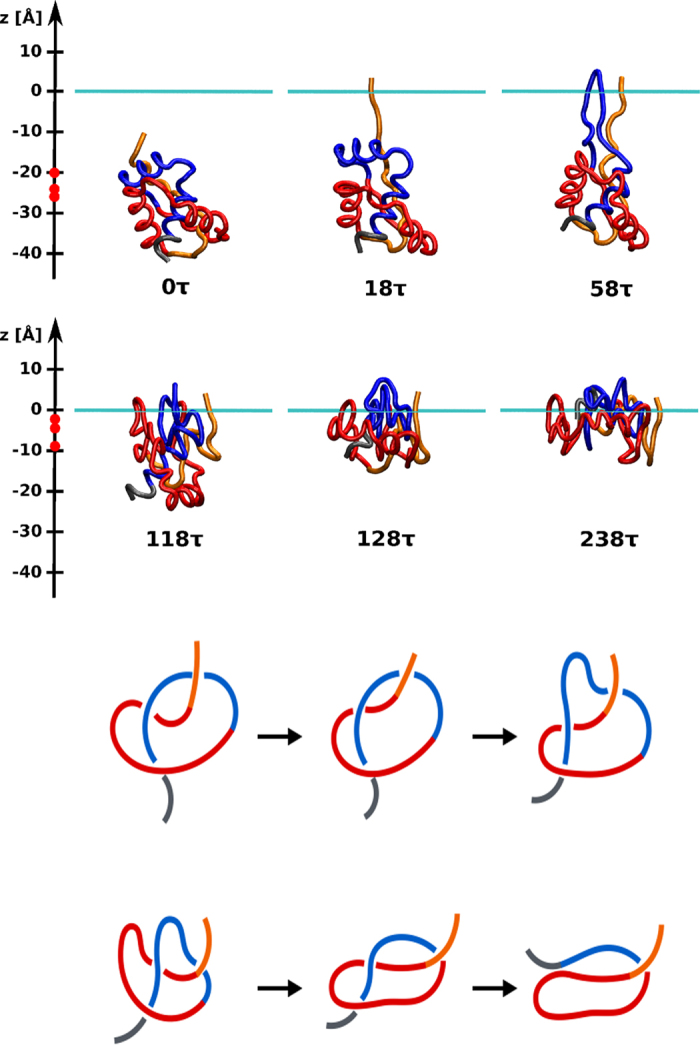
The interface-induced untying of the knot in 2EFV. The 6 top panels show snapshots of the backbone conformations at times indicated. The horizontal line shows the level corresponding to the center of the interface. The first panel corresponds to the protein that is still away from the interface. The red marks on the *z*-xis indicate the subsequent positions of the center of mass of the protein. The 6 bottom panels show the schematic topological representations corresponding to the conformations shown in the top panels. Untying is accomplished through the DT mechanism.

The situation changes for the shallowly knotted proteins. Now the knots do untie. An untying process is illustrated in Fig. 4 (2EFV and two of its mutants), Fig. 5 (2EFV and 4LRV) and Fig. 6 (2EFV). Adsorption of 2EFV is driven by the hydrophobic N-terminus (its first two residues are hydrophobic while the hydropathy indices of the first 10 residues add up to −1.11) but the untying process takes place primarily through DT (7% by MT) at the hydrophilic C-terminus. Due to the distortion of the whole protein, it is difficult to decide whether the unfolding process involves one or two loops so we do not provide the partitioning numbers.

The last nine residues in 2EFV are LNCELVKLD and their hydropathy indices add up to +0.41. However, the protein can tie back again either through DT or SK and hence *P*_*k*_ in Fig. 4 decays to a finite value instead of to zero. Overall, the changes in the topology, as described by *P*_*k*_, depend both on the approach to the interface and on the related loss of the contacts.

We now consider two mutations at the C-terminus in 2EFV. The first mutation replaces the last 9-residue sequential segment by LACALVALA which makes it more hydrophobic – the hydropathy indices add up to +2.81 – and the second mutation, to PNPEPPKPD, makes it hydrophilic – the hydropathy indices add up to −2.49. Figure 4 shows that both mutations enhance the probability of staying knotted but mutation 2 is much more effective in doing so. The hydrophobic C-terminus of the first mutation favors an accelerated adsorption with less time to untie. The hydrophilic C-terminus, on the other hand, gets stuck in the water phase which preserves the knotted topology of the protein. In conclusion, the distribution of the hydrophobicity of a knotted protein is a factor contributing to the untying probabilities at the interface.

A similar behavior is observed for 4LRV (Fig. 5) except that this protein is more likely to stay knotted than 2EFV. The two proteins are quite comparable in their linear size in the native state: the radius of gyration for 4LRV is 13.08 Å, and for 2EFV −12.89 Å. However, they differ in the contact-mediated connectivity significantly: 4LRV has 36% more contacts than 2EFV. This feature makes 4LRV harder to untie than 2EFV. Two examples of the interface-induced unknotting of 4LRV are shown in Fig. S3 in SI, which demonstrates two available untying mechanisms of 4LRV, *i.e.* DT and MT with DT occuring more frequently. SK is not observed in the unknotting of 4LRV, which may be due to the fact that the terminal outer segments of 4LRV are too short to form a slipknot.

### Knot-tying at the air-water interface

**Figure 7:**
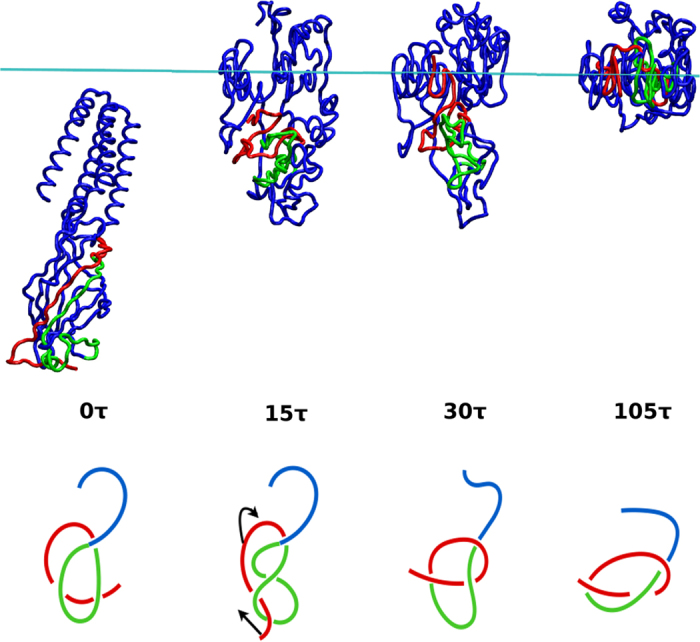
An example of the interface-induced knotting process in 3EAM at *T*_*r*_. The red segment extends from the N-terminus (begins from site 5 since the first four amino acids are not available in the crystal structure) to site 35, the green segment – from 36 to 70, and the blue segment – from 71 to the C-terminus.

If at least one of the terminal segments of an unknotted protein is hydrophobic, there is a possibility that dragging it towards the interface may lead to formation of a knot. This is, in fact, what we found to happen in protein 3EAM with *H* = +0.32. This protein comprises 311 structurally resolved residues. Its native state is unknotted and thermal fluctuations in the absence of the interface do not lead to any knot-tying in the *T*-range between 0.3 and 0.7 *ε*/*k*_*B*_. The net hydropathy score for its N-terminal segment of 8 residues, which should cross an entangled region of this protein to form a knot, is +0.41. Due to the low hydrophobicity of this segment, the knotting process in most trajectories is accomplished when the N-terminus is still in the water phase, as shown in Fig. 7. This terminus gets lifted to the interface together with other segments after the C-terminus (its net hydrophathy of 8 residues is +3.35) of the protein is already adsorbed to the interface.

We find that in 52% of 50 trajectories, a knot forms through DT. An example of a formed trefoil knot is illustrated in Fig. 7. If one makes the N-terminal segment more hydrophobic (to *H* = +2.44) through mutations, then the success in tying the knot is: 68%. If one makes it more polar (to *H* = −1.50) then the success rate is 66%. Thus both mutations increase the knotting probability of 3EAM. The N-terminus of the hydrophobic mutation can be easily adsorbed across the entangled part to the interface, which increases the probability of forming a knot. On the other hand, if the N-terminal segment is made more hydrophilic it may be dragged downward to the water phase after the whole protein gets adsorbed. This phenomenon may create another chance at passing through the entangled part of the protein, increasing the probability of knotting. The knotted conformation need not last – the knot, if very shallow, may untie through the subsequent evolution.

We have also observed knotting in a transport protein 4NYK from *Gallus gallus*. However, we have not detect it in other plausible candidates such as 1A91, 1FJK, 1H54, 1H7D, 1KF6, 1N00, 1NNW, 1O4T, 1RW1, 1YEW, 2EC8, 2FV7, 2HI7, 2I0X, 2IVY, 2OIB, 3JUD, 3KLY and 3KZN. These proteins have been selected so that at least one of their termini is hydrophobic since such terminal segments have an enhanced probability of moving through the protein on approaching the interface.

## Conclusions

We have demonstrated that the forces associated with the air-water interface may affect the topological state of a protein. It is an interesting question to ask how to devise experimental ways to detect such transformations, if they indeed arise. After all, our model is coarse-grained and phenomenological, especially in its account of the interface. Thus, further investigation such as the comparison between atomistic and coarse-grained models would be required. All atom simulations of air-water interfaces, even in the absence of any proteins, are expected be complicated due to the huge number of molecules needed to set up a necessary density profile that would be stationary. Our simple model points to possible topological transformations that may take place at the interface. We hope it will provide motivation for studies by other means.

It should be noted that topological transformations can also occur in the intrinsically disordered proteins simply as a result of time evolution. This has been demonstrated through all-atom simulations for polyglutamine chains of a sufficient length^68^. For 60-residue chains, about 10% of the statistically independent conformations have been found to be knotted. These knots can be shallow or deep and are not necessarily trefoil. The knotted character of these conformation may be related to the toxicity of proteins involved in Huntington disease^69^.

Contrary to the results reported in ref. 66, we find that shallow knots always untie before the unfolding on heating and the untying of deep knots may follow unfolding only at unrealistically high temperatures, though perhaps at acceptable concentration of the denaturant. It should be noted that homopolymers without any attractive contact interactions may tie knots purely entropically.

## Additional Information

**How to cite this article**: Zhao, Y. *et al*. Topological transformations in proteins: effects of heating and proximity of an interface. *Sci. Rep.* **7**, 39851; doi: 10.1038/srep39851 (2017).

**Publisher's note:** Springer Nature remains neutral with regard to jurisdictional claims in published maps and institutional affiliations.

## Supplementary Material

Supplementary Information

## References

[b1] Buck D. DNA topology. Proc. Symp. Appl. Math. 66, 1–33 (2009).

[b2] Marenduzzo D., Micheletti C. & Orlandini E. The knotted strands of life. Physics World 26, 30–34 (2013).

[b3] Arsuaga J. . DNA knots reveal a chiral organization of DNA in phage capsids. Proc. Natl. Acad. Sci. USA 102, 9165–9169 (2005). 15958528 10.1073/pnas.0409323102 PMC1166588

[b4] Krasnow M. A. . Determination of the absolute handedness of knots and catenanes of DNA. Nature 304, 559–560 (1983). 6308470 10.1038/304559a0

[b5] Dean F. B., Stasiak A., Koller T. & Cozzarelli N. R. Duplex DNA knots produced by Escherichia coli topoisomerase I: Structure and requirements for formation. J. Biol. Chem. 260, 4975–4983 (1985). 2985571

[b6] Berger J. M. Type II topoisomerases. Curr. Opin. Struct. Biol. 8, 26–32 (1999). 10.1016/s0959-440x(98)80006-7 9519293

[b7] Stasiak A. & Flamini A. Simulation and action of DNA topoisomerases to investigate boundaries and shapes of spaces of knots. Biophys. J. 87, 2968–2975 (2004). 15326026 10.1529/biophysj.104.045864 PMC1304770

[b8] Wasserman S. A., Dungan J. M. & Cozzarelli N. R. Discovery of a predicted DNA knot substntiates a model for site-specific recombination. Science 229, 171–174 (1985). 2990045 10.1126/science.2990045

[b9] Ernst C. & Sumners D. W. A calculus for rational tangles: application to DNA recombination. Math. Proc. Camb. Phil. Soc. 108, 489–515 (1990).

[b10] Witz G., Dietler G. & Stasiak A. Tightening of DNA knots by supercoiling facilitates their unknotting by type II DNA topoisomerases. Proc. Natl. Acad. Sci. USA 108, 3608–3611 (2011). 21321228 10.1073/pnas.1016150108 PMC3048145

[b11] Taylor W. A deeply knotted protein structure and how it might fold. Nature 406, 916–919 (2000). 10972297 10.1038/35022623

[b12] Mallam A. & Jackson S. Folding studies on a knotted protein. J. Mol. Biol. 346, 1409–1421 (2004). 10.1016/j.jmb.2004.12.055 15713490

[b13] Mallam A. & Jackson S. Probing nature’s knots: The folding pathway of a knotted homodimeric protein. J. Mol. Biol. 359, 1420–1436 (2006). 16787779 10.1016/j.jmb.2006.04.032

[b14] Virnau P., Mirny L. A. & Kardar M. Intricate knots in proteins: function and evolution. Plos. Comp. Biol. 2, e122 (2006). 10.1371/journal.pcbi.0020122 PMC1570178 16978047

[b15] Virnau P., Mallam A. & Jackson S. Structures and folding pathways of topologically knotted proteins. J. Phys. Cond. Mat. 23, 033101–17 (2011). 10.1088/0953-8984/23/3/033101 21406854

[b16] Sułkowska J. I., Rawdon E. K., Millet K. C., Onuchic J. N. & Stasiak A. Conservation of complex knotting and slipknotting patterns in proteins. Proc. Natl. Acad. Sci. USA 109, E1715–E1723 (2012). 22685208 10.1073/pnas.1205918109 PMC3387036

[b17] Mallam A. L. & Jackson S. E. Knot formation in newly translated proteins is spontaneous and accelerated by chaperonins. Nature chemical biology 8(2), 147–153 (2012). 10.1038/nchembio.742 22179065

[b18] Jamroz M. . KnotProt: a database of proteins with knots and slipknots. Nucl. Acid. Res. 43, D306–314 (2015). 10.1093/nar/gku1059 PMC4383900 25361973

[b19] Wallin S., Zeldovich K. B. & Shakhnovich E. I. The folding mechanics of a knotted protein. J. Mol. Biol. 368, 884–893 (2007). 17368671 10.1016/j.jmb.2007.02.035 PMC2692925

[b20] Sułkowska J. I., Sułkowski P. & Onuchic J. N. Dodging the crisis of folding proteins with knots. Proc. Natl. Acad. Sci. USA 106, 3119–3124 (2009). 19211785 10.1073/pnas.0811147106 PMC2651233

[b21] Li W., Terakawa T., Wang W. & Takada S. Energy landscape and multiroute folding of topologically complex proteins adenylate kinase and 2ouf-knot. Proc. Natl. Acad. Sci. USA 109, 17789–17794 (2012). 22753508 10.1073/pnas.1201807109 PMC3497823

[b22] Beccara S. A., Skrbic T., Covino R., Micheletti C. & Faccioli P. Folding pathways of a knotted protein with a realistic atomistic atomic force field. PLOS Comp. Biol. 9, e1003002 (2013). 10.1371/journal.pcbi.1003002 PMC3605060 23555232

[b23] Noel J. K., Onuchic J. N. & Sułkowska J. I. Knotting a protein in explicit solvent. Phys. Chem. Lett. 4, 3570–3573 (2013).

[b24] Chwastyk M. & Cieplak M. Cotranslational folding of deeply knotted proteins. J. Phys. Cond. Matter 27, 354105 (2015). 10.1088/0953-8984/27/35/354105 26292194

[b25] Chwastyk M. & Cieplak M. Multiple folding pathways of proteins with shallow knots and co-translational folding. J. Chem. Phys. 143, 045101 (2015). 26233164 10.1063/1.4927153

[b26] Koniaris K. & Muthukumar M. Knottedness in ring polymers. Phys. Rev. Lett. 66, 2211–2214 (1991). 10043425 10.1103/PhysRevLett.66.2211

[b27] Sułkowska J. I., Sułkowski P., Szymczak P. & Cieplak M. Tighttening of knots in proteins. Phys. Rev. Lett. 100, 058106 (2008). 18352439 10.1103/PhysRevLett.100.058106

[b28] Cieplak M., Allen D. B., Leheny R. L. & Reich D. H. Proteins at air-water interfaces: a coarse-grained approach. Langmuir 30, 12888–96 (2014). 25310625 10.1021/la502465m

[b29] Head J. F., Mealy T. R., McCormack F. X. & Seaton B. A. Crystal structure of trimeric carbohydrate recognition and neck domains of surfactant protein A. J. Biol. Chem. 278, 43254–60 (2003). 12913002 10.1074/jbc.M305628200

[b30] Graham D. E. & Philips M. C. Proteins at liquid interfaces: Kinetics of adsorption and surface denaturation. J. Colloid. Interface Sci. 70, 403–414 (1979).

[b31] Lee M. H., Reich D. H., Stebe K. J. & Leheny R. L. Combined passive and active microrheology study of protein-layer formation at an air-water interface. Langmuir 26, 2650–2658 (2010). 19919016 10.1021/la902881f

[b32] Murray B. S. Rheological properties of protein films. Curr. Opin. Colloid Interface Sci. 16, 27–35 (2011).

[b33] Alonso C., Waring A. & Zasadzinski J. A. Keeping lung surfactant where it belongs: protein regulation of two-dimensional viscosity. Biophys. J. 89, 266–273 (2005). 15833995 10.1529/biophysj.104.052092 PMC1366524

[b34] Proctor G. B., Hamdan S., Carpenter G. H. & Wilde P. A statherin and calcium enriched layer at the air interface of human parotid saliva. Biochem. J. 389, 111–116 (2005). 15769251 10.1042/BJ20042012 PMC1184543

[b35] Euston S. R., Hughes P., Naser M. A. & Westacott R. Molecular dynamics simulation of the cooperative adsorption of barley lipid transfer protein and cis-isocohumulone at the vacuum-water interface. Biomacromolecules 9, 3024–3032 (2008). 18842056 10.1021/bm8004325

[b36] Jordens S. . Adsorption at liquid interfaces induces amyloid fibril bending and ring formation. ACS nano 8, 11071–11079 (2014). 25338060 10.1021/nn504249x

[b37] Berman H. M. . The Protein Data Bank. Nucleic Acids Res. 28, 235–242, www.rcsb.org (2000). 10592235 10.1093/nar/28.1.235 PMC102472

[b38] Lim K. . Structure of the YibK methyltransferase from Haemophilus influenzae (HI0766): a cofactor bound at a site formed by a knot. Proteins 51, 56–67 (2003). 12596263 10.1002/prot.10323

[b39] Bocquet N. . An open-por structure of a bacterial pentameric ligand-gated ion channel. Nature 457, 111–114 (2009). 18987633 10.1038/nature07462

[b40] Cieplak M. & Hoang T. X. Universality classes in folding times of proteins. Biophys. J. 84, 475–488 (2003). 12524300 10.1016/S0006-3495(03)74867-X PMC1302628

[b41] Sułkowska J. I. & Cieplak M. Mechanical stretching of proteins – A theoretical survey of the Protein Data Bank. J. Phys.: Cond. Mat. 19, 283201–60 (2007).

[b42] Sikora M., Sułkowska J. I. & Cieplak M. Mechanical strength of 17 134 model proteins and cysteine spliknots. PLoS Comp. Biol. 5, e1000547 (2009). 10.1371/journal.pcbi.1000547 PMC2759523 19876372

[b43] Wołek K. & Cieplak M. Criteria for folding in structure-based models of proteins. J. Chem. Phys. 144, 185102 (2016). 27179507 10.1063/1.4948783

[b44] Go N. Theoretical studies of protein folding. Annu. Rev. Biophys. Bioeng. 12, 183–210 (1983). 6347038 10.1146/annurev.bb.12.060183.001151

[b45] Takada S. Go-ing for the prediction of protein folding mechanism. Proc. Natl. Acad. Sci. USA 96, 11698–11700 (1999). 10518512 10.1073/pnas.96.21.11698 PMC33792

[b46] Sułkowska J. I. & Cieplak M. Selection of optimal variants of Go-like models of proteins through studies of stretching. Biophys. J. 95, 3174–3191 (2008). 18567634 10.1529/biophysj.107.127233 PMC2547460

[b47] Clementi C., Nymeyer H. & Onuchic J. N. Topological and energetic factors: what determines the structural details of the transition state ensemble and “en-route” intermediates for protein folding? an investigation for small globular proteins. J. Mol. Biol. 298, 937–953 (2000). 10801360 10.1006/jmbi.2000.3693

[b48] Karanicolas J. & Brooks C. L. The origins of asymmetry in the folding transition states of protein L and protein G. Protein Sci. 11, 2351–2361 (2002). 12237457 10.1110/ps.0205402 PMC2373711

[b49] Paci E., Vendruscolo M. & Karplus M. Validity of Go models: Comparison with solvent-shielded empirical energy decomposition. Biophys. J. 83, 3032–3038 (2002). 12496075 10.1016/S0006-3495(02)75308-3 PMC1302383

[b50] Levy Y., Wolynes P. G. & Onuchic J. Protein topology determines binding mechanism. Proc. Natl. Acad. Sci. USA 101, 511–516 (2004). 14694192 10.1073/pnas.2534828100 PMC327178

[b51] Wołek K., Gómez-Sicilia À. & Cieplak M. Determination of contact maps in proteins: a combination of structural and chemical approaches. J. Chem. Phys. 143, 243105–14 (2015). 26723590 10.1063/1.4929599

[b52] Tsai J., Taylor R., Chothia C. & Gerstein M. The packing density in proteins: Standard radii and volumes. J. Mol. Biol. 290, 253–266 (1999). 10388571 10.1006/jmbi.1999.2829

[b53] Hyeon C., Dima R. I. & Thirumalai D. Pathways and kinetic barriers in mechanical unfolding and refolding of RNA an proteins. Structure 14, 1633–1645 (2006). 17098189 10.1016/j.str.2006.09.002

[b54] Valbuena A. . On the remarkable mechanostability of scaffoldins and the mechanical clamp motif. Proc. Natl. Acad. Sci. USA 106, 13791–13796 (2009). 19666489 10.1073/pnas.0813093106 PMC2719556

[b55] Cieplak M., Filipek S., Janovjak H. & Krzysko K. A. Pulling Single Bacteriorhodopsin out of a Membrane: Comparison of simulation and experiment. BBA - Biomembranes 1758, 537–544 (2006). 16678120 10.1016/j.bbamem.2006.03.028

[b56] Szklarczyk O., Staron K. & Cieplak M. Native state dynamics and mechanical properties of human topoisomerase I within a structure-based coarse-grained model. Proteins: Structure, Function and Bioinformatics 77, 420–431 (2009). 10.1002/prot.22450 19452556

[b57] Poma A. B., Chwastyk M. & Cieplak M. Polysaccharide-protein complexes in a coarse-grained model. J. Phys. Chem. B 119, 12028–12041 (2015). 26291477 10.1021/acs.jpcb.5b06141

[b58] Cieplak M. & Robbins M. O. Nanoindentation of virus capsids in a molecular model. J. Chem. Phys. 132, 015101 (2010). 20078182 10.1063/1.3276287

[b59] Cieplak M. & Robbins M. O. Nanoindentation of 35 virus capsids in a molecular model: Relating mechanical properties to structure. PLOS ONE 8, e63640 (2013). 23785395 10.1371/journal.pone.0063640 PMC3681840

[b60] Roos W. H., Bruisma R. & Wuite G. J. L. Physical Virology. Nature Physics 6, 733–743 (2010).

[b61] Kyte J. & Doolittle R. F. A simple method for displaying the hydropathic character of a protein. J. Mol. Biol. 157, 105–32 (1982). 7108955 10.1016/0022-2836(82)90515-0

[b62] Palliser C. C. & Parry D. A. D. Quantitative comparison of the ability of hydropathy scales to recognize surface *β*-strands in proteins. Proteins: Struct. Funct. Gen. 42, 243–255 (2001). 10.1002/1097-0134(20010201)42:2<243::aid-prot120>3.0.co;2-b 11119649

[b63] Lee M. H., Reich D. H., Stebe K. J. & Leheny R. L. Langmuir 26, 2650–2658 (2010). 19919016 10.1021/la902881f

[b64] Cicuta P., Stancik E. J. & Fuller G. G. Shearing or compressing a soft glass in 2D: time-concentration superposition. Phys. Rev. Lett. 90, 236101 (2003). 12857273 10.1103/PhysRevLett.90.236101

[b65] Allan D. B. . Linear and nonlinear microrheology of lysozyme layers forming at the air–water interface. Soft Matt. 10, 7051–60 (2014). 10.1039/c4sm00484a 24969505

[b66] Andrews B. T., Capraro D. T., Sułkowska J. I., Onuchic J. N. & Jennings P. A. Hysteresis as a Marker for Complex, Overlapping landscapes in proteins. J. Phys. Chem. Lett. 4, 180–188 (2012). 23525263 10.1021/jz301893w PMC3601837

[b67] Cieplak M. & Sułkowska J. I. Thermal unfolding of proteins. J. Chem. Phys. 123, 194908 (2005). 16321114 10.1063/1.2121668

[b68] Gómez-Sicilia À., Sikora M., Cieplak M. & Carrión-Vázquez M. An exploration of the universe of polyglutamine structures. PLoS Comp. Biol. 11, e1004541 (2015). 10.1371/journal.pcbi.1004541 PMC4619799 26495838

[b69] Wojciechowski M., Gómez-Sicilia À., Carrión-Vázquez M. & Cieplak M. Unfolding knots by the proteasomes: behavior of folded and neurotoxic proteins. Mol. Biosystems 12, 2700–2712 (2016). 10.1039/c6mb00214e 27425826

