# Supplementary material for: Topological transformations in proteins: effects of heating and proximity of an interface
Source: Sci Rep. 2017 Jan 4;7:39851. doi: 10.1038/srep39851 (PMC5209716; doi:10.1038/srep39851)
Supplement: Supplementary Information [file srep39851-s1.pdf]

Supplementary Information

Topological transformations in proteins: effects of heating and proximity of an interface

Yani Zhao, Mateusz Chwastyk, and Marek Cieplak*
Institute of Physics, Polish Academy of Sciences,
Al. Lotników 32/46, 02-668 Warsaw,
Poland
(Dated: August 22, 2016)

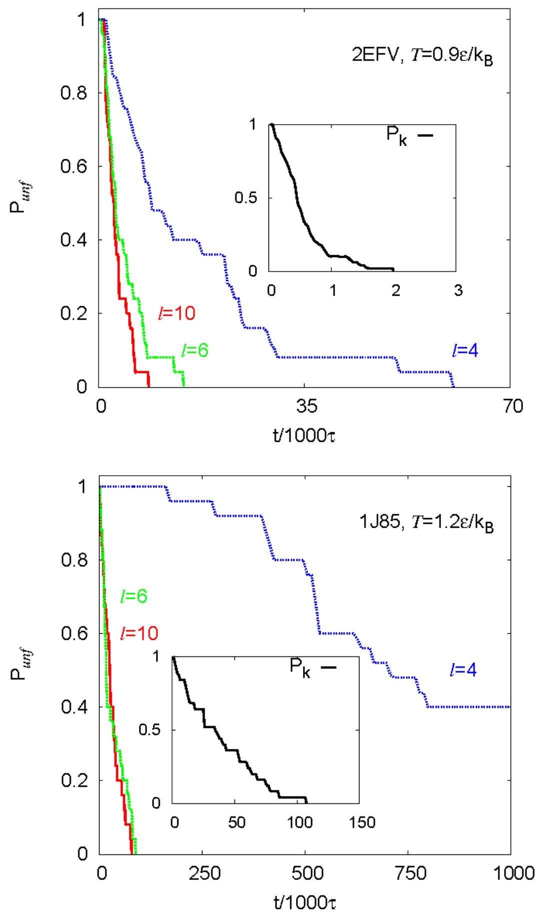

Fig. S 1: The time-evolution of the thermal unfolding probability P_{unf} for the indicated values of l. The top panel is for 2EFV $T = 0.9\ \varepsilon/k_B$ and the bottom panel for 1J85 at $T = 1.2\ \varepsilon/k_B$. The insets show the corresponding time-dependence of P_k. The results are averaged over 50 trajectories.

*Electronic address: `mc@ifpan.edu.pl`

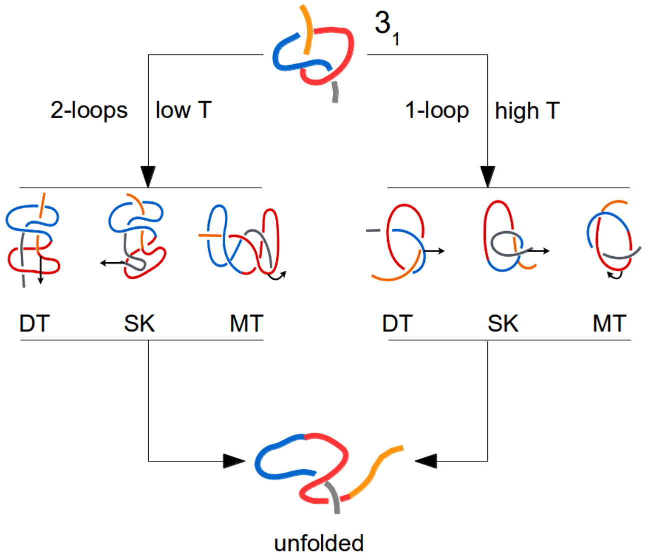

Fig. S 2: The schematic representation of the untying mechanisms of a knotted protein (3_1), in which the N- and C-terminus are colored orange and gray, respectively. The untying process of the protein can be divided into two classes, i. e. the single-loop and the two-loops pathway. The single-loop pathway refers to the situation that only one loop presents during the untying process, and it occurs at high temperatures $T \geq 0.7\varepsilon/k_B$. In this pathway, the protein will be untied via three possible untying mechanisms: DT (direct threading)–one of the terminii threads directly through the loop of the protein, SK (slipknotting)–one terminus of the protein slides through its loop via forming a slipknot, or MT (mousetrap-like mechanism)–the untying of the protein is due to the movement of the loop itself instead of terminus. Yet, two small loops are observed in the two-loops untying process, and it is composed by two stages. In the first stage, one terminus is threaded out of its loop by DT, SK or MT mechanism, but another terminus still remains enveloped in the loop. Next, two small loops vanished under thermal fluctuations after the knot is untied in the first stage. The two-loops pathway is only available for shallow knotted proteins at low temperatures $T \leq 0.7\varepsilon/k_B$.

Table S I: The untying probability P_{unf} and the detailed untying mechanism of deep knot 1J85 at different T in bulk. P_{ref} denotes the probability of retying back from the untied conformation.

T (ε/k_B)	P_{unk} (%)	P_{ref} (%)	1-loop	2-loops
0.9	88	27	68% DT, 32% SK	0%
1.0	100	0	92% DT, 8% SK	0%
1.2	100	0	100% DT, 0% SK	0%

Table S II: The untying probability P_{unf} and the detailed untying mechanism of shallowly knotted proteins 2EFV and 4LRV at different T in bulk. P_{ref} denotes the probability of retying back from the untied conformation.

T (ε/k_B)	2EFV				4LRV			
	P_{unk} (%)	P_{ref} (%)	1-loop	2-loops	P_{unk} (%)	P_{ref} (%)	1-loop	2-loops
0.3	10	100	0%	100% SK	0	–	–	–
0.5	88	93	0%	100% SK	8	100	0%	100% SK
0.7	100	72	34% DT, 32% SK, 6% MT	16% DT, 12% SK, 0% MT	100	84	12% DT, 28% SK, 0% MT	28% DT, 20% SK, 12% MT
1.0	100	0	78% DT, 16% SK, 6% MT	0%	100	0	84% DT, 12% SK, 4% MT	0%
1.2	100	0	76% DT, 16% SK, 8% MT	0%	100	0	84% DT, 16% SK, 0% MT	0%

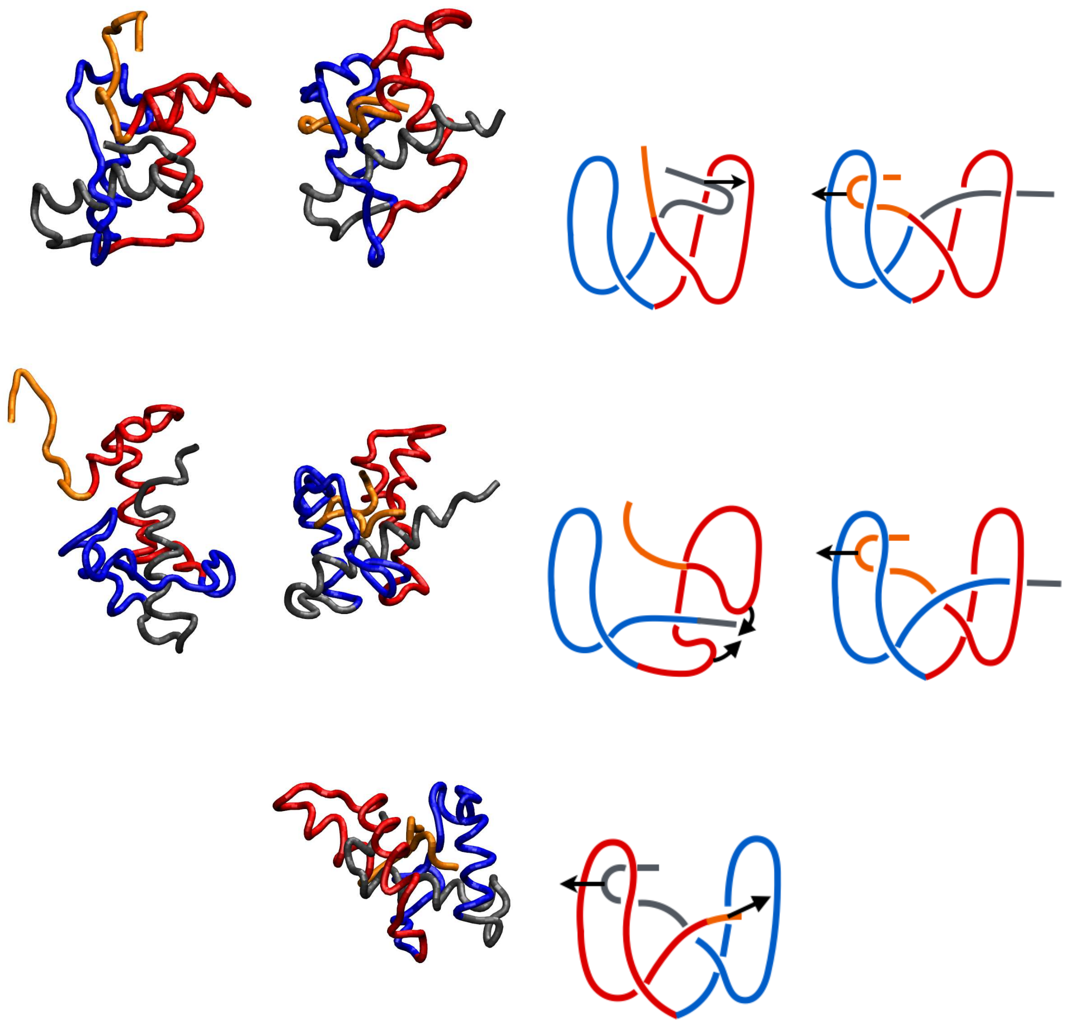

Fig. S 3: Folding mechanisms in 4LRV at T_r. The top, middle, and bottom panels correspond to the SK-SK, EM-SK, and DT-SK pathways respectively. The sites 1-12 are marked in orange, 13-47 in red, 48-81 in blue, and 82-107 in gray.

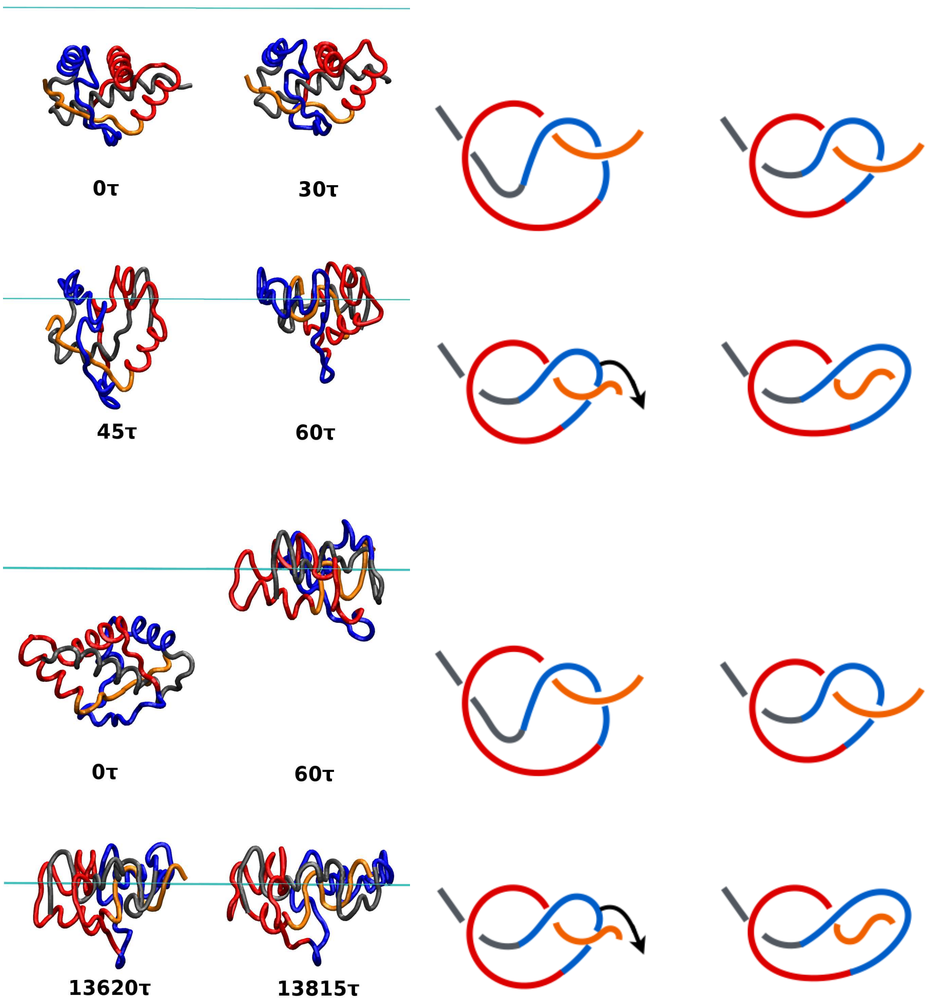

Fig. S 4: Examples of 4LRV untied via DT (top) and MT (bottom) mechanisms at the air-water interface and at $T = T_r$. The color convention is as in Fig. 3.
